# Anionic surfactant reformed carbon composite voltammetric sensor for the efficient and sensitive detection of carmoisine in food samples

**DOI:** 10.1038/s41598-026-58798-z

**Published:** 2026-06-19

**Authors:** Battira M. Sharmila, Jamballi G. Manjunatha, Karnayana P. Moulya, Thiago C. Canevari, Gaber E. Eldesoky

**Affiliations:** 1 Department of Chemistry, FMKMC College, A Constituent College of Mangalore University, Madikeri, Karnataka India; 2 Multifunctional Nanomaterials Hybrid Laboratory (LABNAHM), Engineering School, Mackenzie Presbyterian University, São Paulo, Brazil; 3 Department of Chemistry, College of Science, King Saud University, P.O. Box 2455, Riyadh, 11451 Saudi Arabia

**Keywords:** Anionic surfactant, Azo dye, Carbon nanotubes, Carmoisine, Voltammetric sensor, Chemistry, Environmental sciences, Materials science, Nanoscience and technology

## Abstract

Carmoisine (CM), an azo dye, imparts vibrant red color when added to the food, beverage, cosmetic and pharmaceutical products. The excessive intake of CM causes various health effects and hence it is essential to develop appropriate methods for the determination and quantification of CM in food samples. In this current work, a rapid detection and analysis of CM was performed via cyclic voltammetry (CV) and differential pulse voltammetry (DPV) techniques by harnessing sodium dodecyl sulfate modified composite sensor (SDSMCS). The morphological features and elemental composition of bare carbon nanotube graphite composite sensor (BCNGCS) and SDSMCS surfaces were compared and the difference between the working sensors was assessed. The active surface area was computed as 0.031 cm^2^ for BCNGCS and 0.049 cm^2^ for SDSMCS surfaces by employing CV technique. Key insights into the charge transfer process involved in the oxidation of CM was obtained by electrochemical impedance spectroscopy (EIS). The modified sensor demonstrated improved electrochemical features at optimal experimental conditions. The analysis validated that the electro oxidation of CM proceeds through adsorption-controlled kinetics at the surface of SDSMCS and exhibited a significantly low limit of detection (LOD) of 0.009 µM by CV and 0.073 µM by DPV technique, in the linear range of 0.1 µM to 1.1 µM and 0.1 µM to 0.9 µM, respectively. The attributes like reproducibility and repeatability of the developed SDSMCS were evaluated and the respective relative standard deviation (RSD) of 4.95% and 4.90% verified that the fabricated sensor is reliable for detecting CM in various commercial food samples with a recovery rate exceeding 97%. Surfactant- reformed carbon-based sensors provide a cost-effective and reliable platform that aligns with the evolving trends in field of electrochemical sensing.

## Introduction

**Fig. 1:**
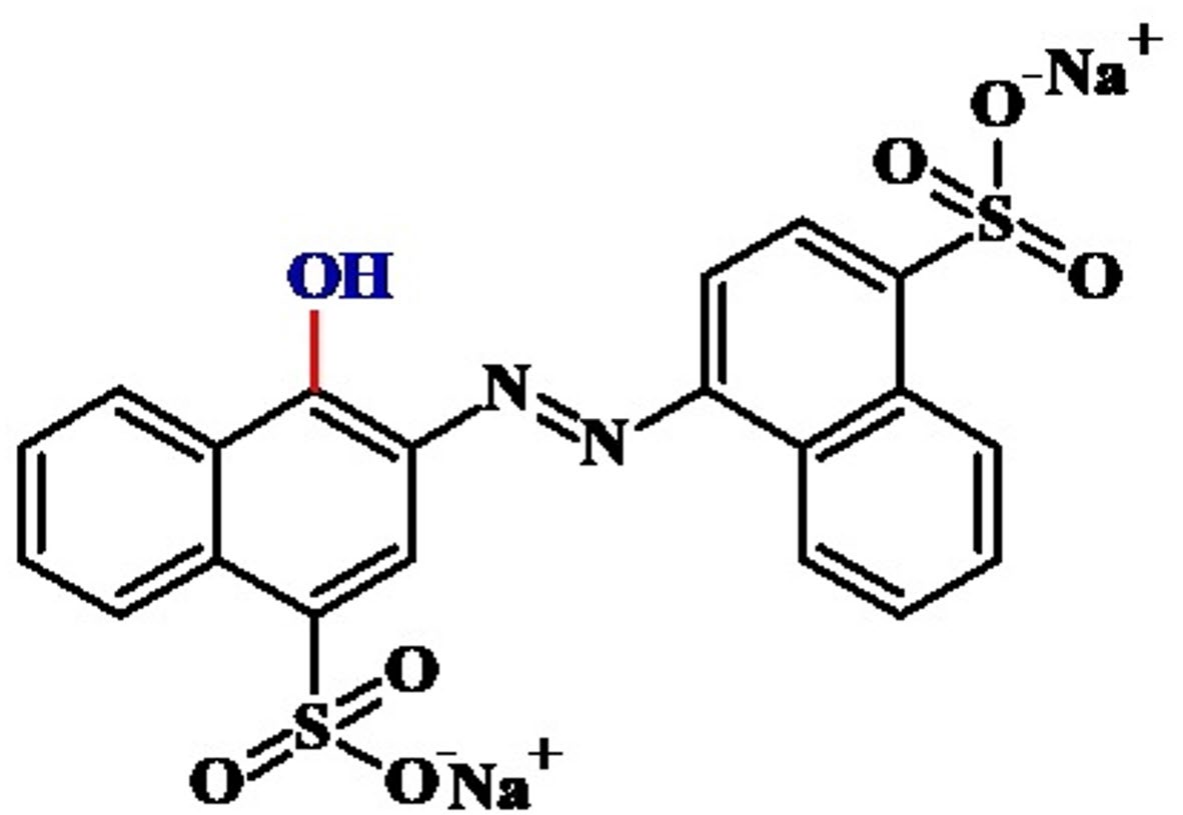
Structure of CM.

Dyes or colorants refer to the compounds that give color to materials like polymers, textiles, food stuff etc. Natural dyes which are extracted from natural sources are eco-friendly, affordable, non- toxic whereas synthetic colorants pose threat to human health and environment along with certain advantages like fastness, being available in various color range and user friendliness^1^. However, the significant stability of the latter towards light, pH and oxygen has made them an unfailing constituent in the food, textile, paper, pharmacological, agrochemical and cosmetic industries^2^. Chemically, CM is a disodium salt with two naphthalene subunits as shown in Fig. 1. It is also known as azorubine and acid red 14 with Europe number (E number) of E122 and available in red or maroon powdered form^3,4^. Obtained by the azo coupling reaction, CM finds its wide application in food, drug and cosmetic industries^5,6^. Studies have reported that these colorants impose ill effects on human health due to their potential toxic nature^7–9^. The daily intake of CM has been limited to 4 mg per kilogram of a person’s body weight, as per Food and Agriculture Organization (FAO) and World Health Organization (WHO) regulations^10^. Over consumption of CM causes variations in renal and hepatic parameters, thus inducing the formation of free radicals causing oxidative stress^11^. Recent reports suggest that CM, when ingested even in low doses, can change the biochemical markers and cause adverse effects on vital organs. The effect becomes more pronounced at high doses and also on long term exposure to low doses^12^. Several methods like spectrophotometry, chromatography, flow injection analysis and chemiluminescence have been developed so far to detect the presence of CM in food items^13–17^. However, the dominant demerits like expensive instrumentation and complex procedures restrict the use of these sophisticated techniques. Electrochemical analysis has emerged as a promising, low- cost and hassle -free approach and it involves redox reactions occurring at the interface of the sensor and the electrolyte^18^. Voltammetry is the widely used technique for the determination of electrochemical features of electrode systems modified in various ways^19^. This technique is highly sensitive, precise and depends on the voltage current for the detection and quantification of electroactive species^20,21^.

Surfactants are amphiphilic compounds with a hydrophobic head and a hydrophilic tail^22,23^. These readily get adsorbed on the surface of the sensor and augment its detection efficiency at the solution and the sensor interface^24,25^. SDS is an anionic surface-active agent which when adsorbed on the sensor surface, enhances the electrochemical performance of the sensor by significantly interacting with the electro active species present in the solution. The micelle forming feature of SDS molecules form stable aggregates on the sensor surface. The electrostatic force of attraction between these micelles and the electro active species increases the concentration of the analyte in the vicinity of the sensor surface which boosts the electron transfer rate, thus intensifying the selective and sensitive detection of the analytes^26,27^. The sensors fabricated by electrode materials like graphite powder (GP) and carbon nanotubes (CNT) display a high surface area with dynamic range of porosity, thus increasing the accessibility of the electrolytes to the internal surface area^28^. GP possesses numerous positive features such as great conductivity, affordability, chemical inertness and hence serves as a versatile electrode material by offering low background current and wide potential windows in voltammetric analysis. The CNT exhibit exceptional qualities such as high chemical stability, excellent conductivity and remarkable catalytic properties as the tube matrices encourage the electron transfer process to a significant extent^29–31^. In this work, CV and DPV techniques have been employed due to their rapid response, simplicity and the ability to convey adequate information about the kinetics of the electrochemical processes^32,33^. This novel approach to detect CM in food samples using a sensor comprising of composite electrode materials reformed with surfactant has provided excellent outcome proving the reliability of this fabricated sensor in availing the relevant applications.

## Experimental methods

### Instrumentation

The analysis was performed with CHI-6038E (electrochemical analyzer from CH Instruments, USA) consisting of working electrodes (BCNGCS and SDSMCS), a counter electrode (platinum wire) and a reference electrode (calomel electrode). The morphology and elemental composition of the working electrodes was assessed by scanning electron microscopy (SEM) and energy-dispersive X-ray spectroscopy (EDXS) at Vigyan Bhavan, University of Mysore, India. The required buffer solutions were prepared using a digital pH meter (EQ-610) procured from Equiptronics, India. Double distilled water (DW) for the preparation of the various solutions was obtained from VITSIL-VBSD/ VBDD water distillation unit.

### Reagents and chemicals

CM (C_20_H_12_N_2_Na_2_O_7_S_2_, 98% pure) and SDS (C_12_H_25_NaSO_4_, 97% pure) were procured from Otto Chemie Pvt. Ltd, Mumbai and Isochem laboratories, Kerala, respectively. CNT (diameter of 110–170 nm, length of 5–9 micron and 90% assay) were obtained from Sigma - Aldrich chemicals. 90% pure silicone oil was procured from Molychem Chemical Manufacturers. 90% pure GP, 99.5% pure potassium chloride (KCl), 98.5% pure potassium ferrocyanide K_4_[Fe(CN)_6_], 99% pure monosodium dihydrogen phosphate (NaH_2_PO_4_) and disodium hydrogen phosphate (Na_2_HPO_4_) were obtained from Nice Chemicals located in Kerala. Chemicals and reagents used in the experimental procedures were of analytical quality and were utilized without any additional purification.

### Preparation of the working sensors

A quantified amount of GP and CNT were blended with silicone oil (acts as a binder) in the ratio of 70:30 to attain a uniform paste. The 3 mm cavity of the teflon tube was methodically filled with the blended paste. To obtain an even surface, the filled end of the teflon tube was gently wiped on a tissue paper. Electrical connection was established by implanting a copper wire through the teflon tube. Throughout the analysis, this fabricated electrode is used as BCNGCS. To achieve the modified sensor for the electro analysis, BCNGCS surface was treated with SDS by drop coating technique and allowed to stand for 5 min for the adequate adsorption of SDS on the sensor surface. The reformed SDSMCS surface was then rinsed with DW to eliminate the excess SDS if any.

### Real sample preparation

Food samples containing CM such as candies and food color were purchased from local grocer to utilize in the analysis. The food samples were mixed thoroughly in distilled water and allowed to settle for 30 min for the complete dissolution of the dye. The supernatant solution was filtered and mixed with appropriate amount of phosphate buffer solution (PBS) for the analysis.

## Results and discussions

### Morphological interpretation of working sensors

**Fig. 2:**
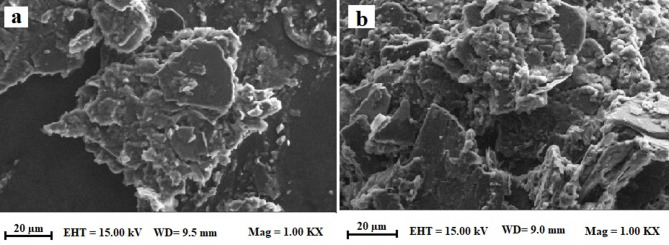
(**a**) SEM image of BCNGCS, (**b**) SEM image of SDSMCS.

The morphological features of the sensor surfaces were assessed by SEM as an effort to correlate the voltammetric performance of the sensor to the surface modification. The images depicted in Fig. 2(a) and Fig. 2(b) exhibits disparity between the two functioning sensors. BCNGCS displays the rough surface which serves as a platform for the adequate deposition of the surfactant during modification. SDSMCS reveals the uniform accumulation of SDS on the surface and the accretion of the surfactant aids in elevating the electrochemical performance of the sensor resulting in the sensitive detection of CM.

**Fig. 3:**
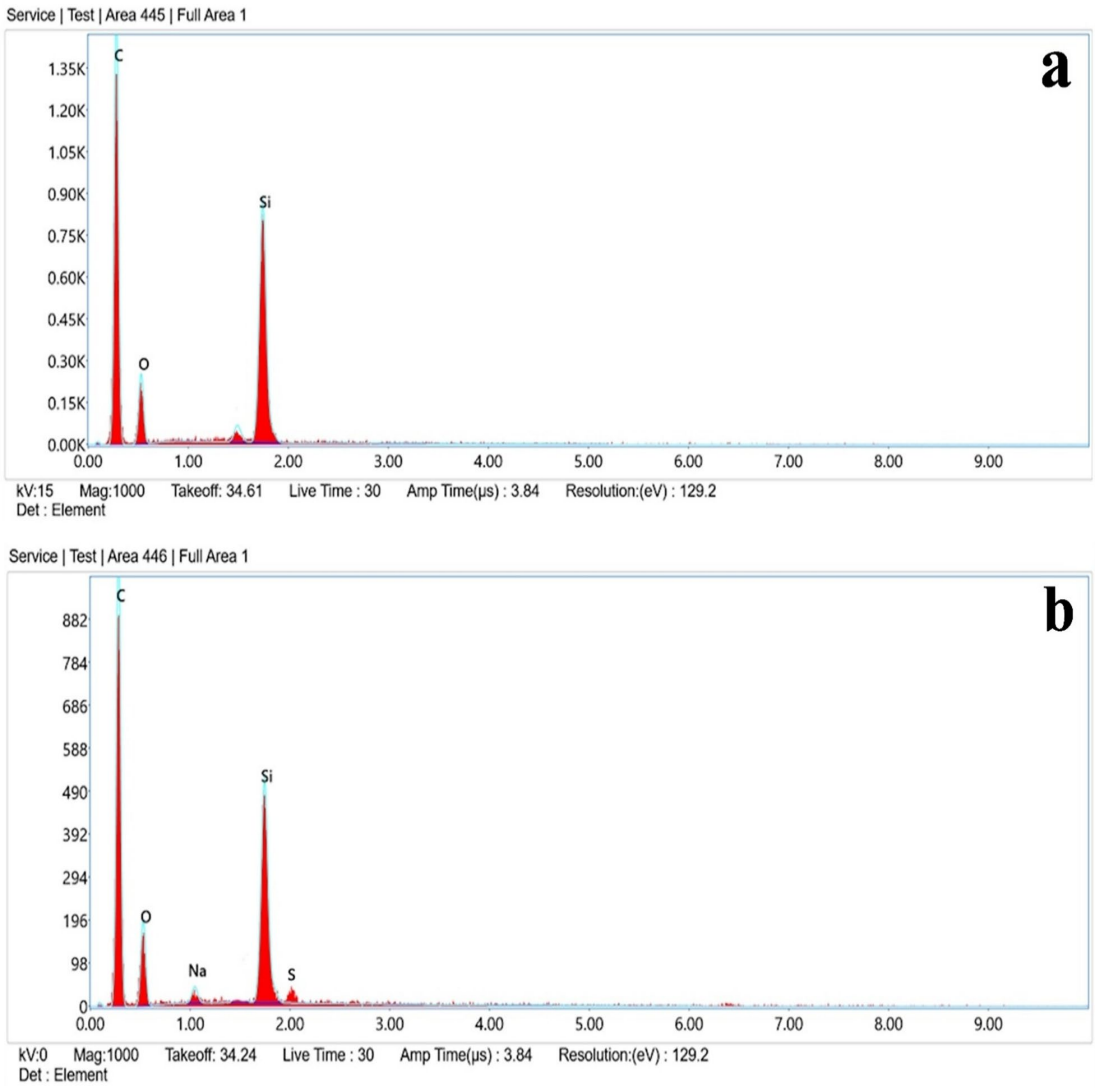
EDXS images **(a)** BCNGCS and **(b)** SDSMCS depicting the elemental composition.

Furthermore, the elemental composition of BCNGCS and SDSMCS were assessed by EDXS. As illustrated in Fig. 3(a) the presence of 72.5% of carbon, 14.4% of oxygen, 12.2% of silicon in BCNGCS is confirmed. Similarly, Fig. 3(b) discloses the existence of 70.5% of carbon, 16% of oxygen, 10.6% of silicon, 1.1% of sodium and 1.2% of sulphur in SDSMCS. The disparity in the elemental composition of BCNGCS and SDSMCS confirms the deposition of SDS on the modified sensor surface.

### EIS study and surface area calculation

**Fig. 4:**
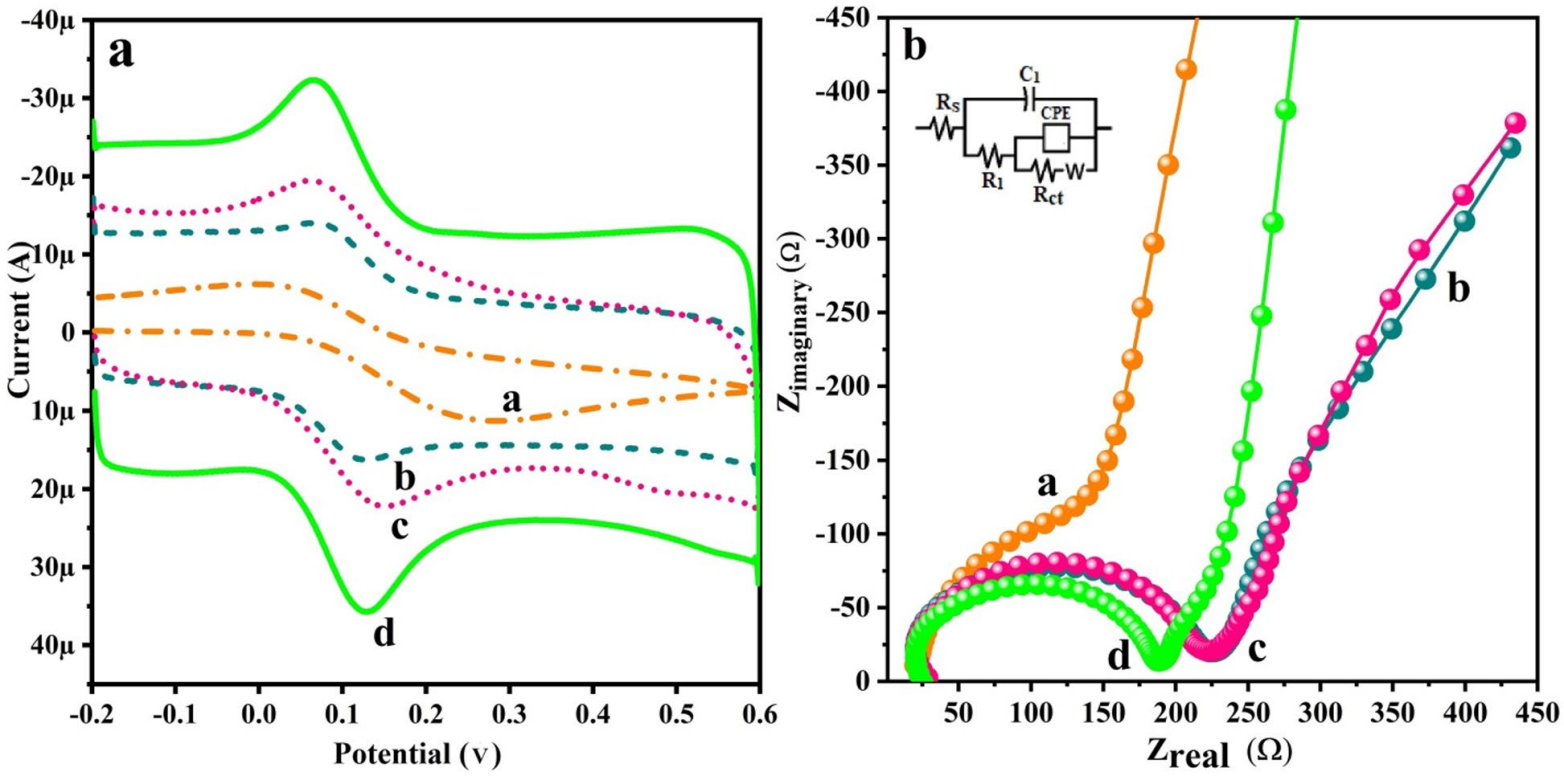
(**a**) CV response for GP (curve a), CNT (curve b), BCNGCS (curve c) and SDSMCS (curve d) (**b**) Corresponding Nquist plot with equivalent circuit.

CV analysis for K_4_[Fe(CN)_6_] (1.0 mM) in 0.1 M KCl (supporting electrolyte) was performed at the surface of various electrode materials and the anodic peak current responses obtained is displayed in Fig. 4(a) (curve ‘a’, ‘b’, ‘c’ and ‘d’ for GP, CNT, BCNGCS and SDSMCS, respectively).

It is apparent from the analysis that the composite electrode material and surfactant modification has enhanced the peak current significantly due to the advancement in the electro active surface area and hence the surface-active sites. With a purpose to validate the same, the electro active surface area of GP, CNT, BCNGCS and SDSMCS were calculated to be 0.015 cm^2^, 0.022 cm^2^, 0.031 cm^2^ and 0.049 cm^2^, respectively by substituting the obtained anodic peak current values in Randles - Sevcik equation,

1 $${\mathrm{I}}_{{{\mathrm{pa}}}} = {\text{ 2}}.{\mathrm{69}} \times {\mathrm{1}}0^{{\mathrm{5}}} {\text{A n}}^{{{\mathrm{3}}/{\mathrm{2}}}} {\mathrm{C}}_{0} {\mathrm{v}}^{{{\mathrm{1}}/{\mathrm{2}}}} {\mathrm{D}}^{{{\mathrm{1}}/{\mathrm{2}}}}$$

Here, I_p.a._, A, n, C_0_, ν and D designates anodic peak current, electro active surface area, number of electrons (here *n* = 1), concentration of K_4_[Fe(CN)_6_, scan rate and diffusion co-efficient (7.6 × 10^− 6^ cm^2^s^− 1^), respectively. The variation in active surface area of the electrode materials validates that the SDS deposition has caused raise in the active surface area of SDSMCS which has apparently increased the active sites causing an enhanced electrochemical activity.

EIS provides mechanistic and kinetic data of the electrochemical processes which is essential to interpret the efficiency of the fabricated sensor^34^. As a redox probe, 1.0 mM K_4_[Fe(CN)_6_] in 0.1 M KCl was subjected to EIS at the functioning sensor surfaces and the achieved outcomes are represented in Fig. 4(b). The EIS data were fitted to a Randles equivalent circuit in which R_s,_ R_1,_ C_1_, R_ct_, W and CPE signify the solution resistance, resistance, capacitance, charge transfer resistance, Warburg impedance and constant phase element. The Nquist plot displays semi-circles at higher frequencies and linear portions (due to the presence of W) at the lower frequencies. The semi-circles are of dissimilar radii for GP (curve a), CNT (curve b), BCNGCS (curve c) and SDSMCS (curve d) and the Warburg element confirms diffusion of the redox probe. The R_ct_ values observed for GP, CNT, BCNGCS and SDSMCS were 307 Ω, 221 Ω, 222 Ω and 187 Ω, respectively. The variance in the Rct values of different electrode materials reflects the difference in their electron transfer kinetics. The smaller semi-circle with lower R_ct_ value (187 Ω) obtained for SDSMCS validates the influence of the modifier (SDS) in the enhancement of the electron transfer kinetics due to amplified electro- active surface area resulting in increased conductivity.

### Optimization of the sensor modifier

**Fig. 5:**
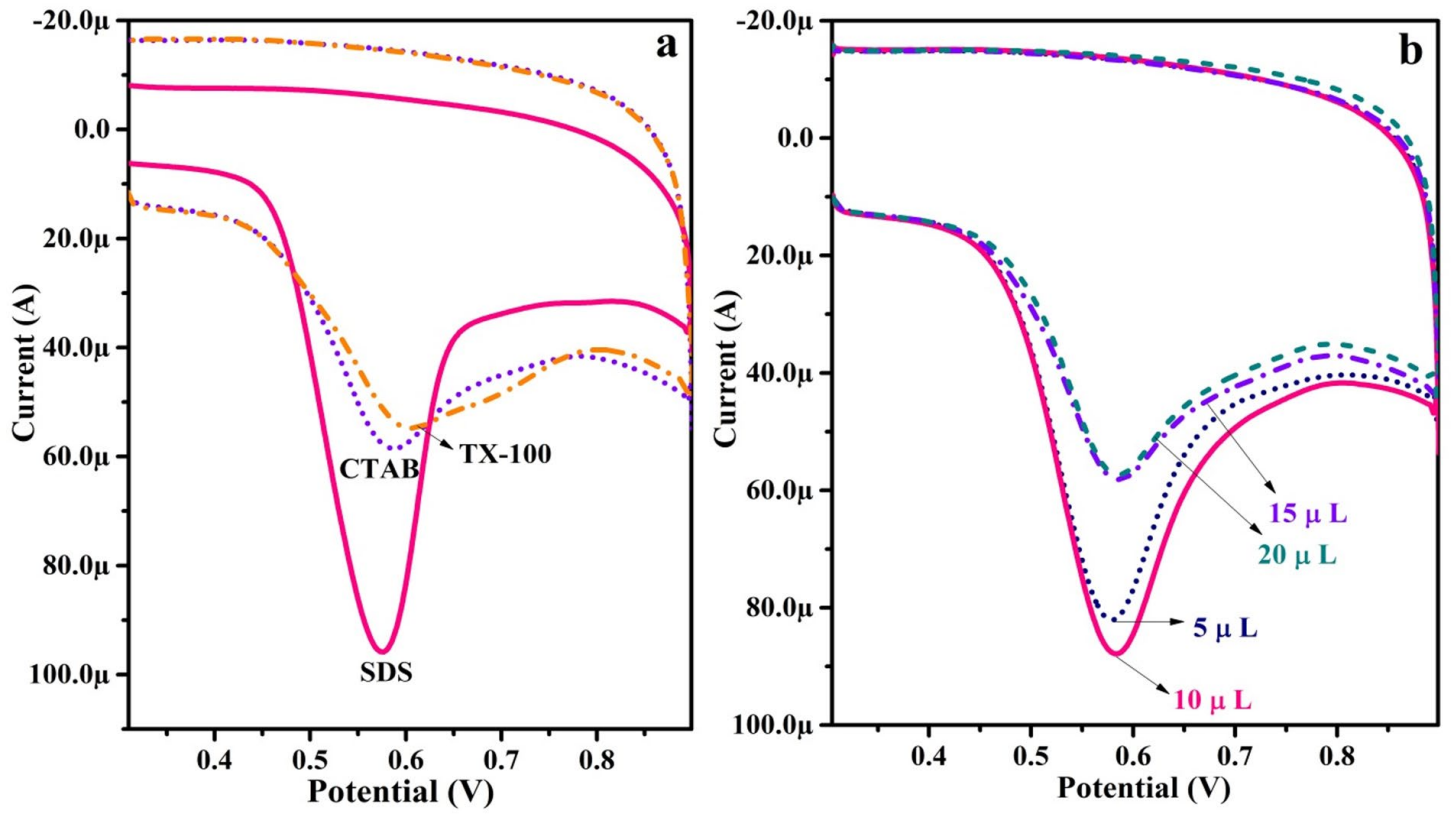
CV representation of (**a**) 1.0 mM CM at BCNGCS surfaces modified with CTAB, TX-100 and SDS, (**b**) 1.0 mM CM at BCNGCS surfaces modified with different volume of SDS.

The electrochemical performance of the SDSMCS altered when different surface modifiers were used in the analysis. The optimization of the modifier in order to achieve maximum voltammetric response was performed using CV technique. The current responses of 1.0 mM CM at the three different BCNGCS surfaces modified with surfactants (using drop coating technique) such as cetyltrimethylammonium bromide (CTAB), Triton X-100 (TX-100) and SDS were recorded and compared as depicted in Fig. 5(a). Among these, SDS modified sensor responded with a high peak current response of 96.13 µA for CM in PBS (0.2 M) at a potential window of 0.3–0.9 V. The maximum current response can be accredited to the favorable interface offered by SDS for the CM oxidation at the sensor surface. In addition, the appropriate amount of surfactant for drop coating method in order to attain maximum electrochemical activity was evaluated. CV was recorded for 1.0 mM CM with BCNGCS modified with SDS in four different volumes of 5 µL, 10 µL, 15 µL and 20 µL. The observed current responses are displayed in Fig. 5(b) which discloses that 10 µL of SDS drop coated on BCNGCS yields a stable peak with significantly high peak current value of 88.22 µA. The oxidation peak current augmented when the amount of SDS was increased from 5 µL to 10 µL and further increase in the volume (15 µL and 20 µL) resulted in the diminishing of the peak current responses. This variation is due to the immobilization of the sensor surface in the presence of excess SDS^35^. Hence, for further investigation of CM, 10 µL of SDS was considered for the sensor modification.

### Optimization of accumulation time and potential

**Fig. 6:**
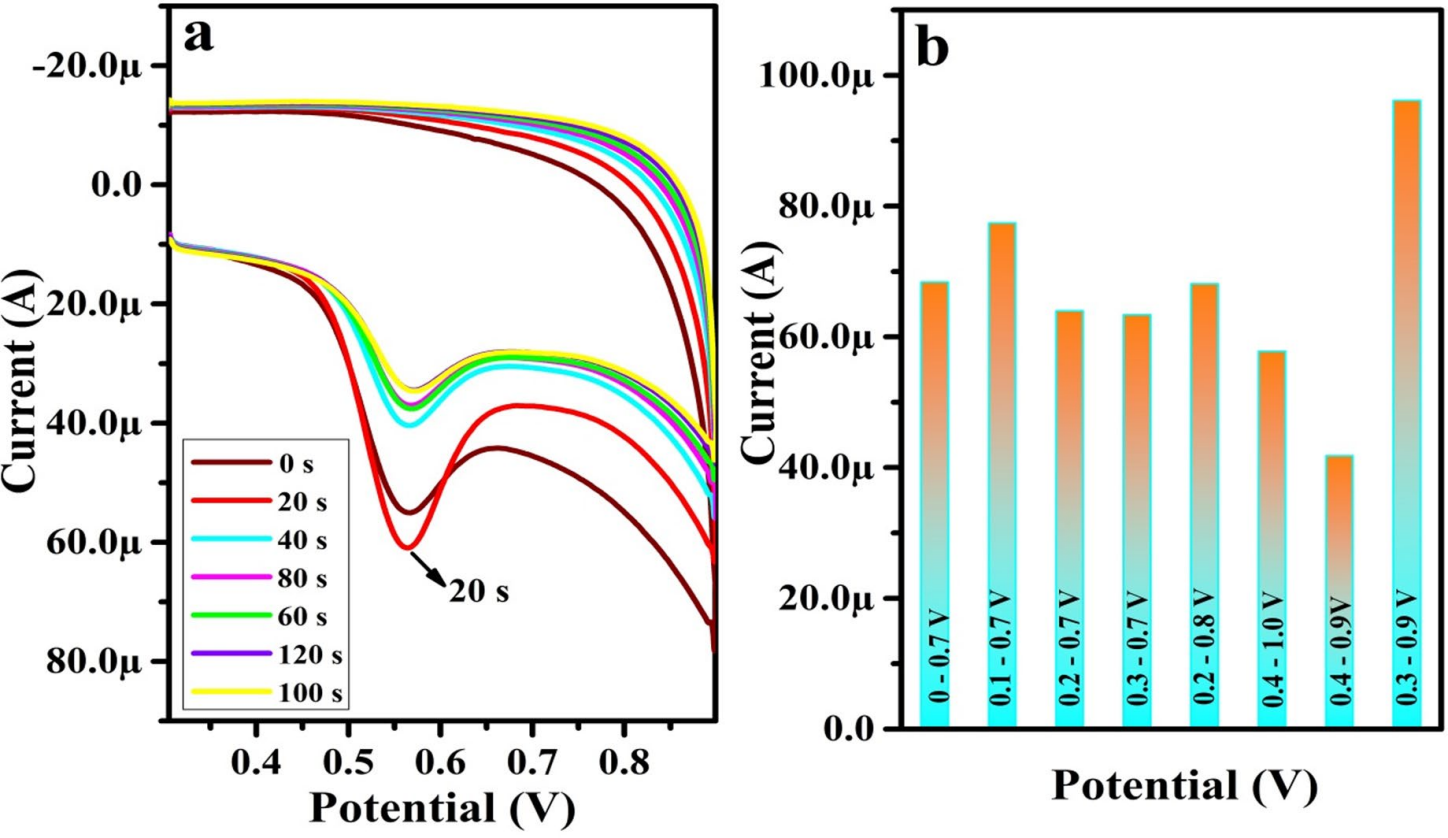
(**a**) CV depiction of 1.0 mM CM at SDSMCS surface at different time intervals, (**b**) anodic peak current responses 1.0 mM CM at SDSMCS surface at different potential window.

The time required for the analyte to get adsorbed on the surface of the sensor greatly influences the sensitivity of the peak current. As represented in Fig. 6(a), CV was recorded for 1.0 mM CM at SDSMCS surface at different time intervals (0 s to 120 s) at 0.1 V/s scan rate in PBS (0.2 M). It is apparent that the 20 s exhibits an oxidation peak with higher peak current value, which signifies that the adequate accumulation of CM on the sensor surface occurs at 20 s. Hence, during the analysis the accumulation time of 20 s was maintained to achieve efficient analytical results. Furthermore, the potential window was optimized by recording CV in various potential gaps as represented in Fig. 6(b). Evidently, the peak current value is high at potential window of 0.3–0.9 V in comparison with the other potential ranges, owing to the elevated electrochemical activity of CM at SDSMCS surface. Hence, the potential gap of 0.3 –0.9 V was considered for the voltammetric measurements in the analysis.

### Electrochemical behavior of CM at the functional sensor surfaces

**Fig. 7:**
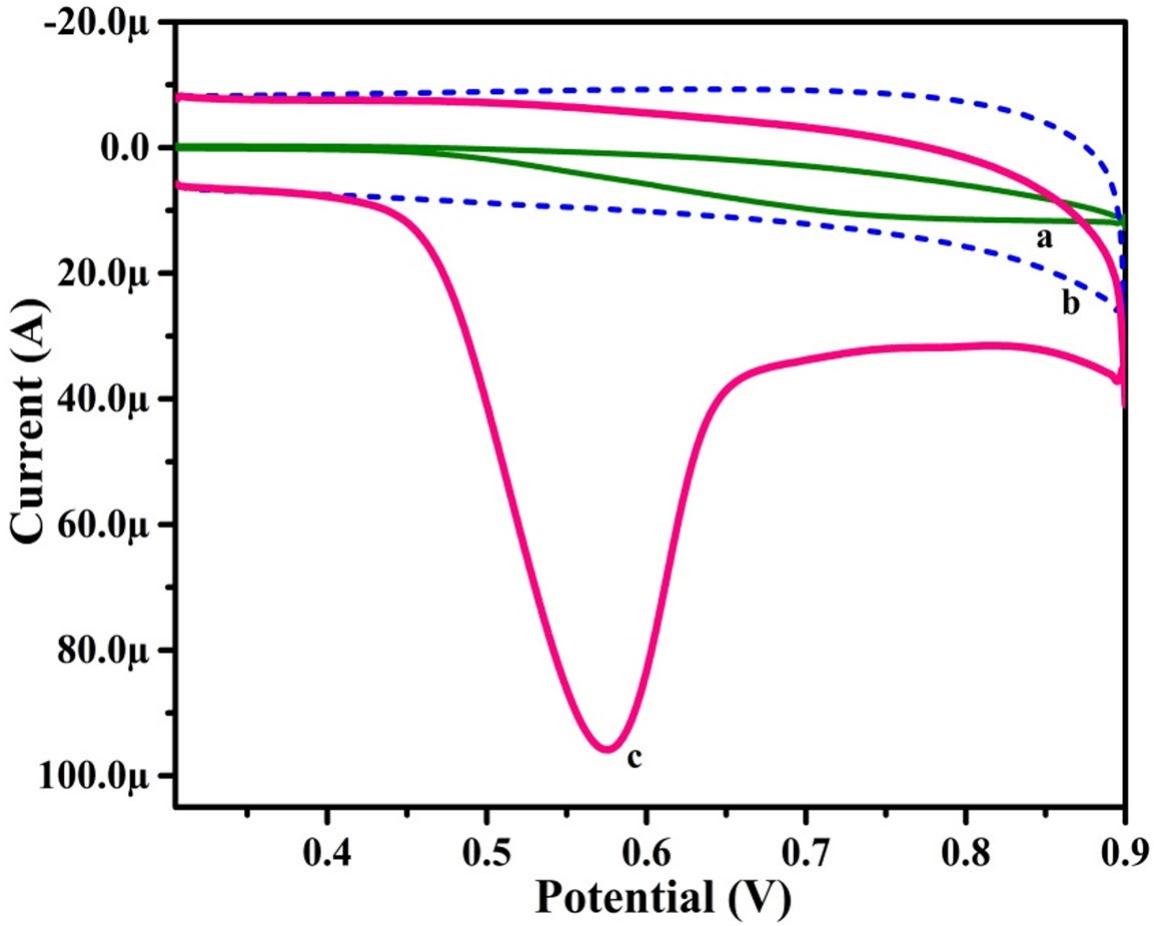
CV representation of 1.0 mM CM in 0.2 M PBS at BCNGCS (curve a), SDSMCS (curve c) surfaces, only PBS at SDSMCS surface (curve b).

The electrochemical activity of CM at BCNGCS and SDSMCS surfaces was inspected via CV technique. As demonstrated in Fig. 7, the voltammetric measurements were made for 1.0 mM CM at 0.1 V/s scan rate in PBS (0.2 M) at BCNGCS and SDSMCS surfaces (curve ‘a’ and curve ‘c’, respectively). The curve ‘b’ corresponds to the CV response of 0.2 M PBS in the absence of CM. The anodic current response at BCNGCS is feeble (10.63 µA at an anodic peak potential of 0.7917 V which is in converse with the significant current response of CM (96.13 µA at a peak potential of 0.5762 V) at SDSMCS surface. An obvious variation in the oxidation current responses of BCNGCS and SDSMCS is observed and the enhancement in the electrochemical activity can be attributed to the surfactant modification of the sensor.

### Study of the impact of electrolytic pH

**Fig. 8:**
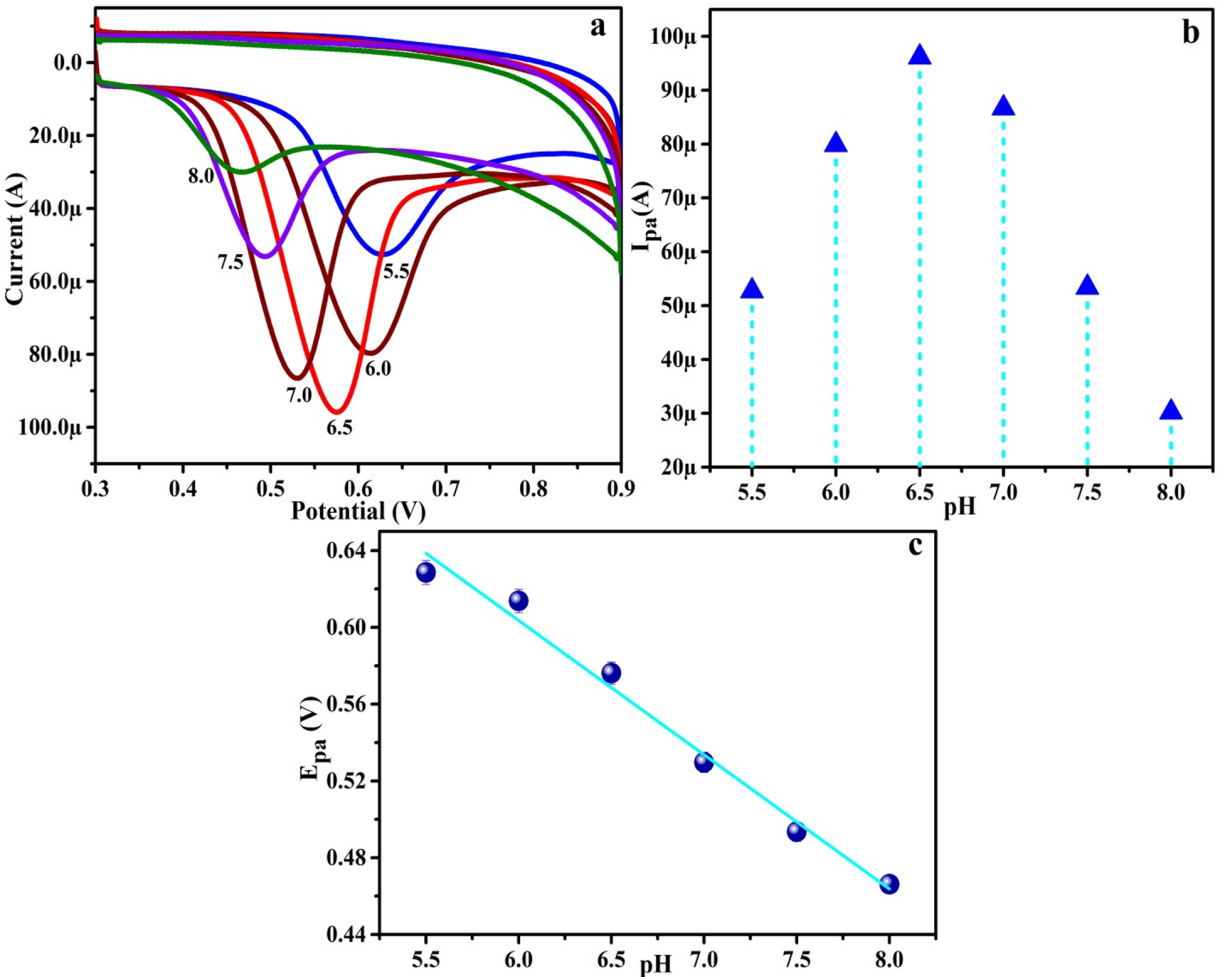
(**a**) CV of CM (1.0 mM) in PBS (0.2 M) of varied pH, (**b**) I_p.a._ vs. pH plot and (**c**) E_p.a._ vs. pH plot.

The pH of the supporting electrolyte greatly influences the electrochemical activity of CM at SDSMCS surface and it is essential to optimize the pH for further analyses. CV was documented for 1.0 mM CM in 0.2 M PBS of varied pH (5.5 to 8.0) and as illustrated in Fig. 8(a), it was observed that the oxidation peak current fluctuates with varying pH. It is evident from the Fig. 8 (b) (anodic peak current, I_p.a._ versus pH plot) that at pH of 6.5, SDSMCS exhibits high peak current value of 96.38 µA and at other pH values, the current response subsides. The optimum pH value in which the maximum electrochemical activity is observed is 6.5 and for further subsequent analyses, the PBS of pH 6.5 was considered. The negative linear shift observed in the plot of E_p.a._ (anodic peak potential) versus pH has been represented in Fig. 8(c). The linearity can be expressed in the linear regression equation (LRE),

2 $${\mathrm{E}}_{{{\mathrm{pa}}}} \left( {\mathrm{V}} \right)\, = \,{\mathrm{1}}.0{\mathrm{22}}{-}0.0{\text{69 pH }}\left( {{\mathrm{V}}/{\mathrm{pH}}} \right){\text{ R}}^{{\mathrm{2}}} = {\text{ }}0.{\mathrm{9928}}$$

The slope of 0.069 (approximately nearer to the theoretical value 0.059) in Eq. (2) conveys that electrochemical oxidation of CM at SDSMCS, involves same number of electrons and protons.

### Effect of scan rate

**Fig. 9:**
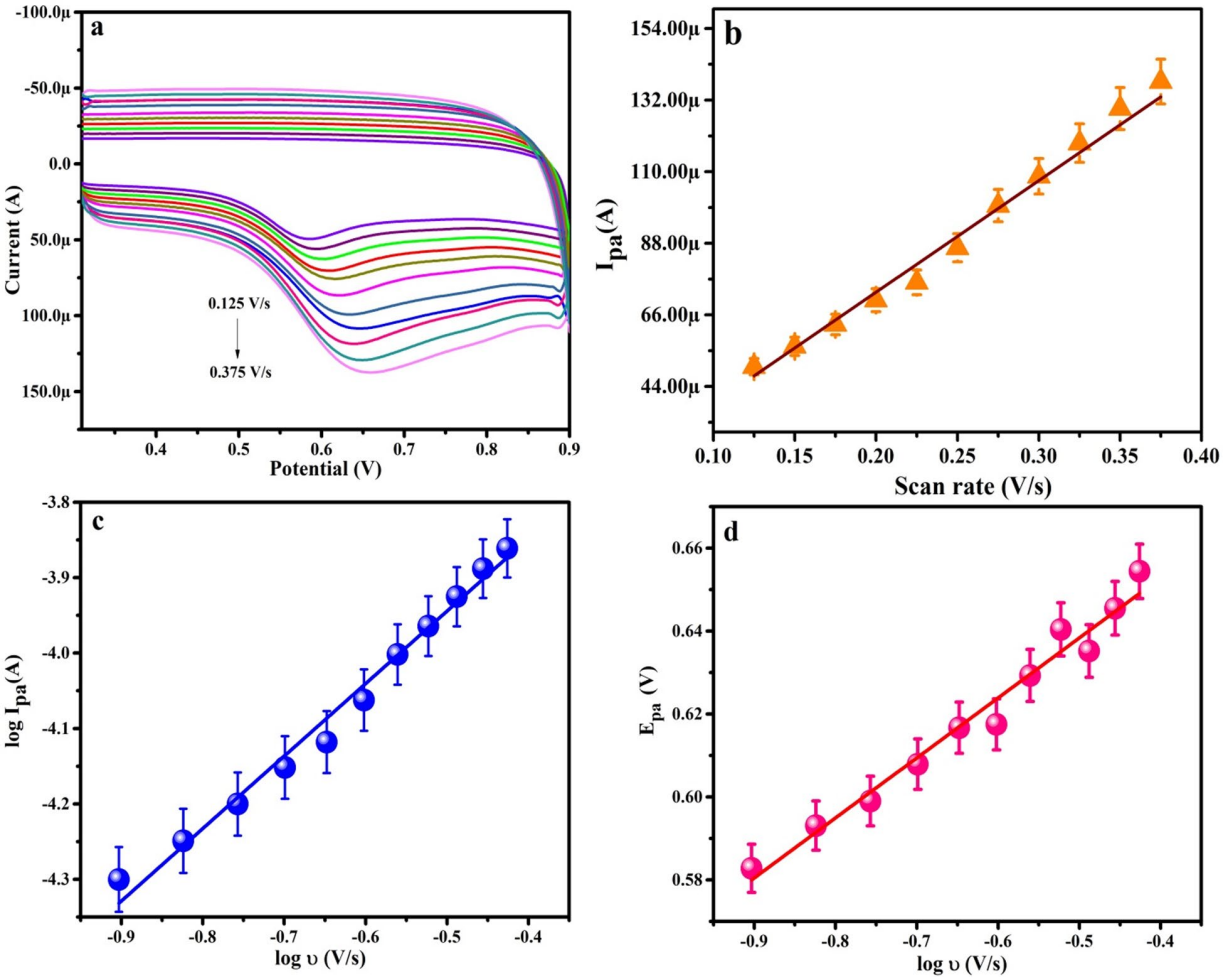
(**a**) CV response of CM in 0.2 M PBS (pH 6.5) at varied scan rate, (**b**) plot of I_p.a._ vs. scan rate (ν), (**c**) graph of log I_p.a._ versus log ν and (**d**) plot of E_p.a._ versus log ν.

CV was employed to assess the effect of scan rate on oxidation peak current and oxidation peak potential during the CM detection. The analysis was performed for 1.0 mM CM in 0.2 M PBS at varied scan rate ranging from 0.125 to 0.375 V/s and the outcome of the CV analysis has been illustrated in Fig. 9(a). As the scan rate is augmented from 0.125 to 0.375 V/s, oxidation peak current shift towards positive potential as depicted in the Fig. 9(b), indicating that the oxidation process is kinetically controlled. The absence of cathodic peak signifies that the process is irreversible^36^. A fair linearity was observed between the plot of I_p.a._ vs. scan rate which can be expressed in LRE as:

3 $${\mathrm{I}}_{{{\mathrm{pa}}}} \left( {\mathrm{A}} \right)\, = \,{\mathrm{2}}.{\mathrm{27}} \times {\mathrm{1}}0^{{ - \,{\mathrm{8}}}} + {\text{ 3}}.{\mathrm{63}} \times {\mathrm{1}}0^{{ - \,{\mathrm{4}}}} v\left( {{\mathrm{V}}/{\mathrm{s}}} \right);{\text{ R}}^{{\mathrm{2}}} \, = \,0.{\mathrm{9952}}$$

Further, the linearity obtained from the plot of log of I_p.a._ vs. log of scan rate (ν) as represented in Fig. 9(c), can be expressed in LRE as below:

4 $${\text{log I}}_{{{\mathrm{pa}}}} \left( {\mathrm{A}} \right)\, = \,{\mathrm{3}}.{\mathrm{47}}\, + \,0.{\text{96 log v}}\left( {{\mathrm{V}}/{\mathrm{s}}} \right);{\text{ R}}^{{\mathrm{2}}} = {\text{ }}0.{\mathrm{9929}}$$

The linearity observed in Eq. (3) with regression coefficient R^2^ = 0.9952 and a slope of 0.96 (≈ 1) obtained in Eq. (4) validates that CM oxidation progresses through adsorption-controlled kinetics at SDSMCS surface. The number of electrons involved in the electro oxidation process was evaluated by obtaining necessary data from plot of E_p.a._ vs. log of scan rate as portrayed in Fig. 9(d). The LRE can be represented as –

5 $${\mathrm{E}}_{{{\mathrm{pa}}}} \left( {\mathrm{V}} \right)\, = \,0.{\mathrm{7114}}\, + \,0.{\text{1457 log v}}\left( {{\mathrm{V}}/{\mathrm{s}}} \right);{\text{ R}}^{{\mathrm{2}}} \, = \,0.{\mathrm{987}}0$$

Substituting the slope from Eq. (5) in Laviron’s equation, the number of electrons was enumerated as 1.18 (≈ 1).

6 $${\mathrm{E}}_{{{\mathrm{pa}}}} = {\text{ E}}^{0} + {\text{ }}[{\mathrm{2}}.{\mathrm{3}}0{\text{3 RT}}/\alpha {\mathrm{nF}}\left] {{\text{ log }}} \right[{\mathrm{RTk}}^{0} /\alpha {\mathrm{nF}}\left] {{\text{ }} + {\text{ }}} \right[{\mathrm{2}}.{\mathrm{3}}0{\text{3 RT}}/\alpha {\mathrm{nF}}]{\text{ log v}}$$

Here, ‘R’, ‘T’, ‘α’, ‘n’, ‘F’, ‘k^0’^ signifies universal gas constant, absolute temperature, charge transfer co-efficient, number of electrons, Faraday’s constant and heterogeneous rate constant, respectively.

**Fig. 10:**
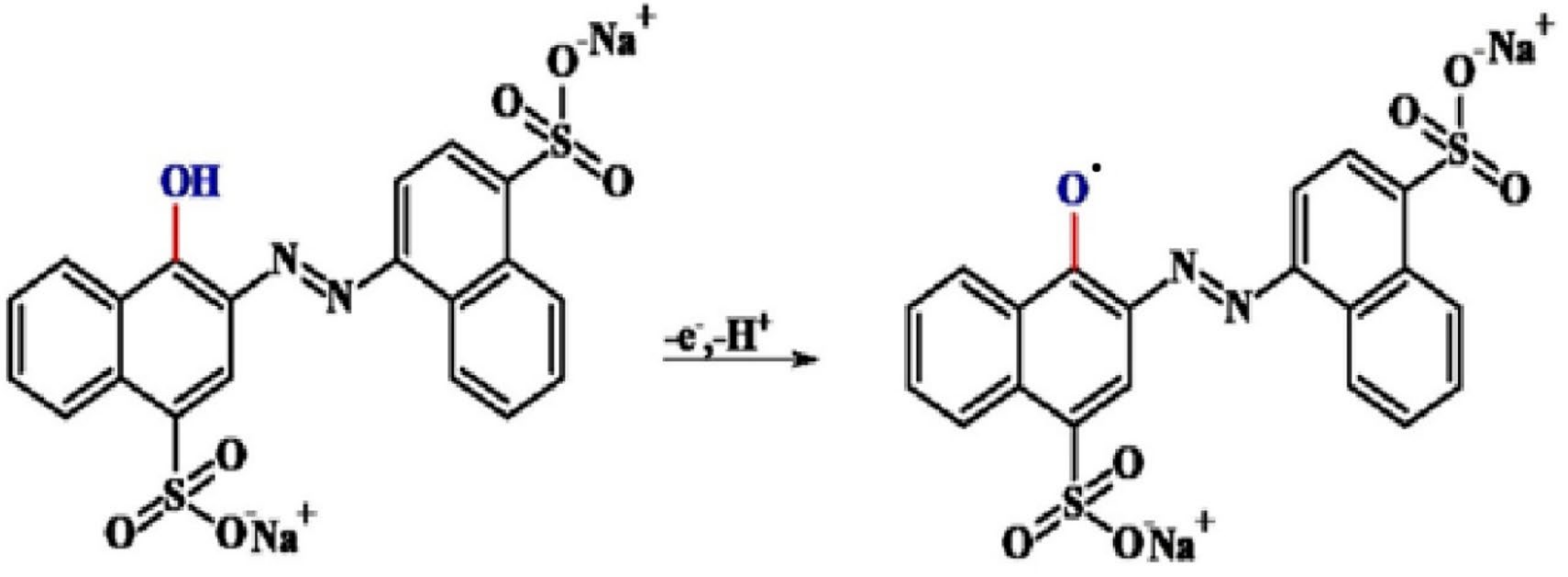
CM oxidation on SDSMCS surface.

The feasible mechanism of adsorption-controlled electro-oxidation of CM at SDSMCS surface occurring with the transference of one electron has been illustrated in Fig. 10. At the surface of SDSMCS, the naphthol moiety of CM undergoes single electron oxidation to an unstable phenoxy radical intermediate. Furthermore, this intermediate undergoes rearrangement (C-N bond) and oxidation (azo group) leading to the oxidative destruction of the molecule. This mechanism accounts for the occurrence of single irreversible oxidation peak in the CV analysis^37–39^.

### Impact of CM concentration on oxidation peak current

**Fig. 11:**
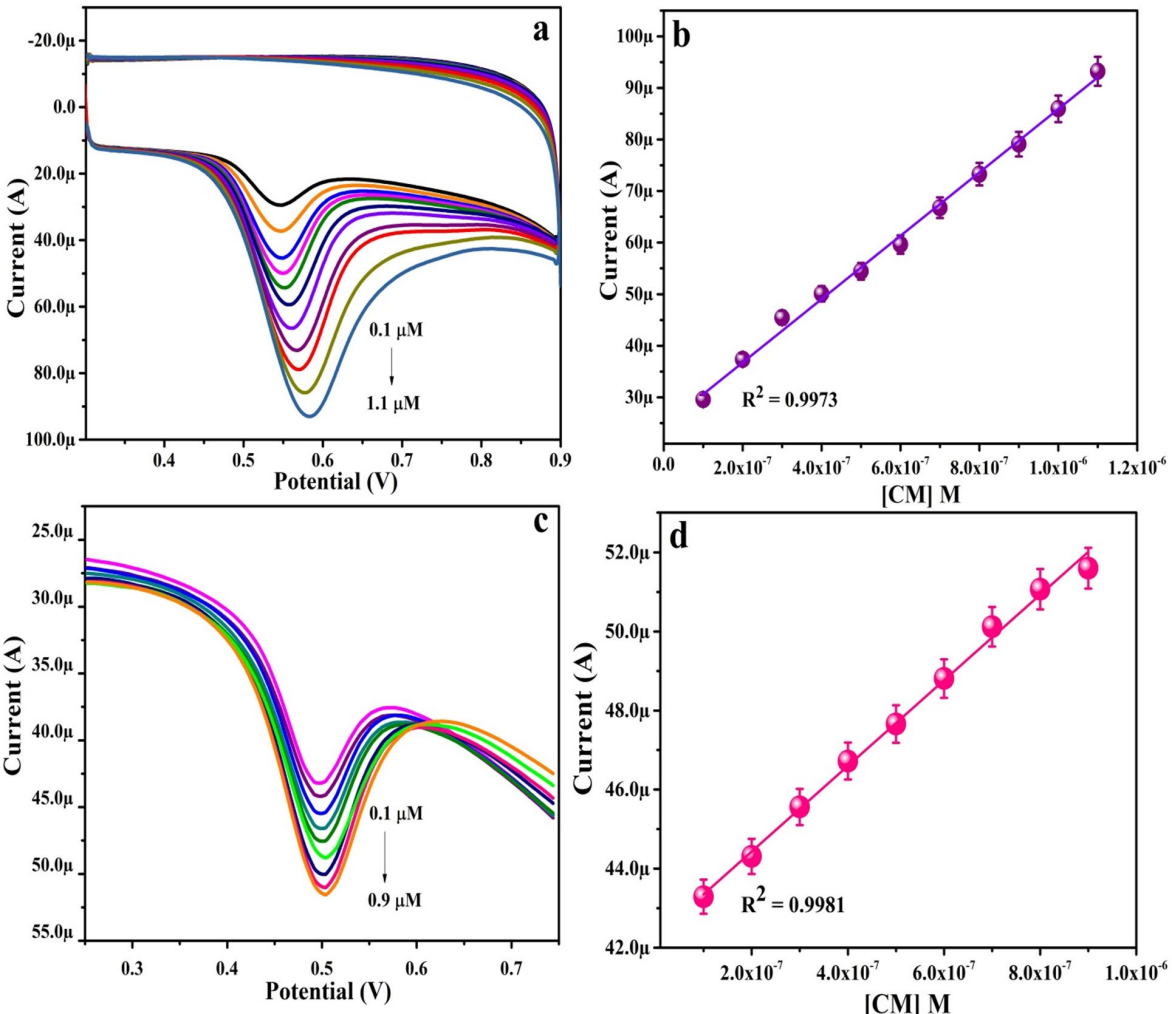
CV and DPV depiction of oxidation peak current enhancement with varying concentration (Fig. 11(a) and 11(c), respectively), Calibration plots representing the linear relationship of oxidation current and diverse CM concentration (Fig. 11(b) and 11(d), respectively for CV and DPV).

With a purpose to assess the sensitivity of the fabricated SDSMCS in detection and quantification of CM, techniques such as CV and DPV were performed for various concentrations of CM in 0.2 M PBS. From Fig. 11(a) and 11(c), the linear upsurge of the anodic peak current with the varied CM concentration (0.1 µM to 1.1 µM in CV and 0.1 µM to 0.9 µM in DPV) can be observed. The slope values obtained from the calibration plots Fig. 11(b) and 11(d) were applied in the expressions, LOQ = 10 S/N and LOD=3 S/N to get respective LOQ and LOD values. Here, ‘S’ and ‘N’ denote the standard deviation of blank and slope of calibration plots. Accordingly, the values obtained were LOD = 0.009 µM and LOQ = 0.031 µM by CV technique and LOD = 0.073 µM and LOQ = 0.244 µM by DPV technique. When these values were compared with the values reported in earlier works (as illustrated in Table 1), the present work provided a considerably low LOD and LOQ, substantiating the superior sensitive attribute of the fabricated SDSMCS.

**Table 1:** LOD values of related works.

Technique	Electrode	Linear range	LOD	Reference
DPV	GO-Fe_3_O_4_-G_4_ PAMAM/ILCPE	0.1–170	0.02 µM	^40^
SQV	CPE/1-M-3BIBr/NiO/CNTs	70–650	0.02 µM	^41^
Spectrophotometry	-	0.02–3.50 µg mL^− 1^	0.017 µg mL^− 1^	^42^
CV	GCE	0.05–0.5 mg/L	0.02 mg/L	^43^
CV	GCE modified with CTAB	0.2–2.0 mg/dm^3^	0.1 mg/dm^3^	^44^
DPV	MoS_2_ NSs/ SPE	0.1–400 µM	0.03 µM	^45^
DPV	ZnO HQSs - SPGE	0.08–190 µM	0.02 µM	^46^
ASV	HMDE	0.03–0.9 µM	0.004 µM	^47^
DPV	g-C3N4/ILs/CPE	0.4–125 µM	0.1 µM	^48^
DPV	CPT- BDDE	0.059–1.31 µmolL^− 1^	7.0 nmolL^− 1^	^49^
CV/ DPV	SDSMCS	0.1 µM to 1.1 µM / 0.1 µM to 0.9 µM	0.009 µM /0.073 µM	Present work

GO-Fe_3_O_4_-G_4_ PAMAM/ILCPE - graphene oxide Fe_3_O_4_ fourth generation poly (amidoamine) ionic liquid modified carbon paste electrode.

SQV- Square wave voltammetry.

CPE/1-M-3BIBr/NiO/CNTs - carbon paste and NiO/CNTs composite modified with 1-methyl-3-butylimidazolium bromide.

GCE - glassy carbon electrode.

CTAB - cetyltrimethylammonium bromide.

MoS_2_ NSs/ SPE - screen printed electrode modified with molybdenum disulfide nanosheets.

ZnO HQSs - SPGE - screen printed electrode modified with zinc oxide hollow quasi- spheres.

ASV - adsorptive stripping voltammetry.

HMDE - hanging mercury drop electrode.

g-C3N4/ILs/CPE - graphitic carbon nitride/1-butyl-3-methylimidazolium hexafluorophosphate ionic liquids modified carbon paste electrode.

CPT- BDDE - Cathodically pretreated boron - doped diamond electrode.

### Applicability of SDSMCS in real samples

**Fig. 12:**
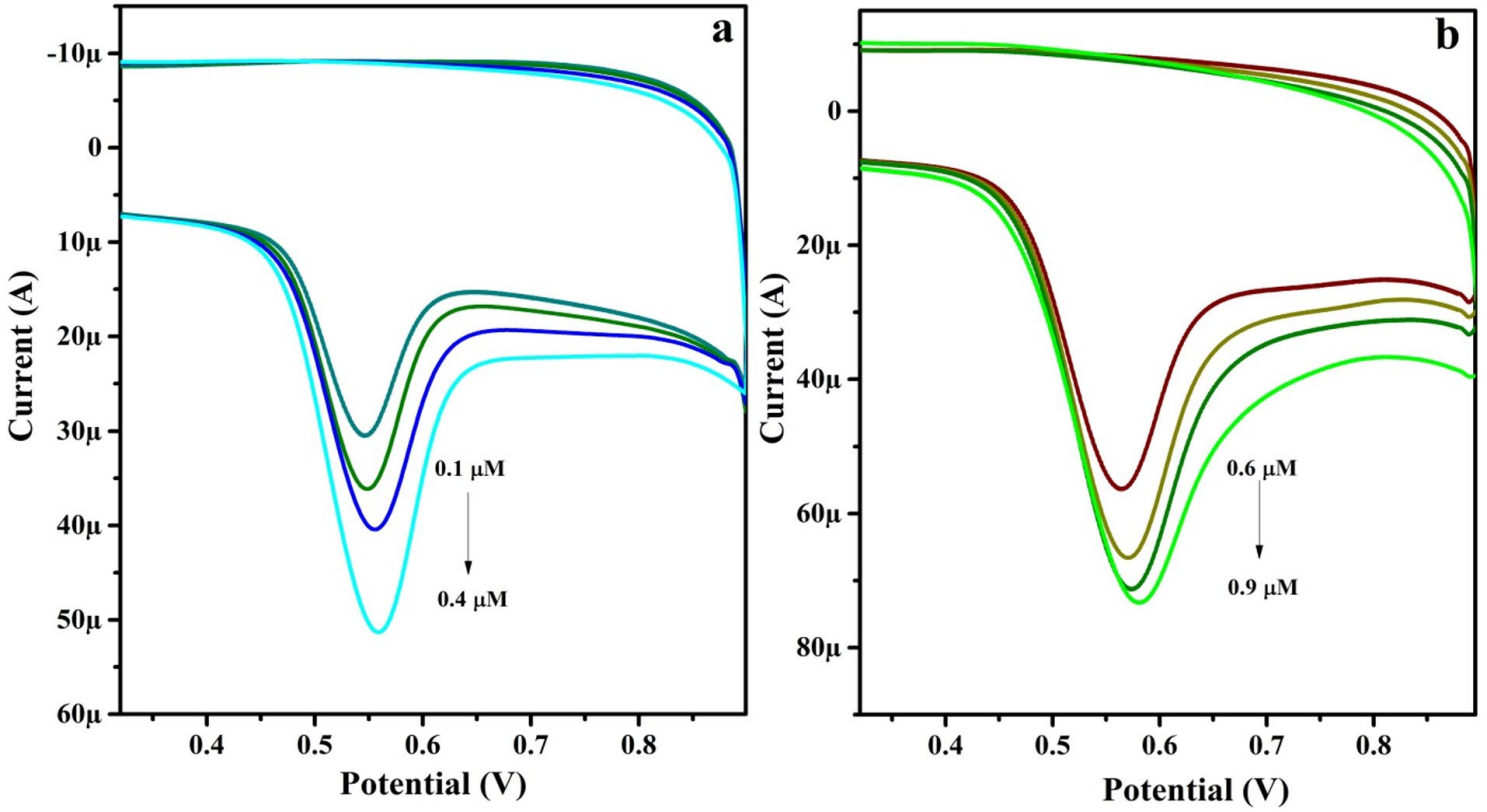
CV responses of (**a**) candies and (**b**) food color in 0.2 M PBS at a scan rate of 0.1 V/s.

The application of the developed sensor in practical samples was investigated via CV technique. The formulated solution of food color and gems were subjected to the analysis and the current responses for the concentration ranging from 0.1 µM to 0.4 µM and 0.6 µM to 0.9 µM (for candies and food color are demonstrated in Fig. 12(a) and 12(b), correspondingly.

From the data gained from the analysis, the recovery rates were calculated and the outcome was > 97% as illustrated in Table 2. From this analysis, it is apparent that the developed SDSMCS is highly efficient in detecting CM in food samples with considerable recovery rates. Additionally, this substantial outcome can be accredited to the highly selective and reliable voltammetric technique employed in the analysis. In comparison with chromatographic and spectrophotometric techniques, CV is less vulnerable to matrix effects as the detection is based on the selectivity limited to the electro- active species undergoing redox reactions at the applied potential, rather than retention time or absorbance^50^.

**Table 2:** Recovery rates of food samples.

Sample	Added (µM)	Found (µM)	Recovery %
**Candies**	0.1	0.097	97.10
	0.2	0.195	97.59
	0.3	0.296	98.81
	0.4	0.389	97.34
**Food color**	0.6	0.588	98.04
	0.7	0.690	98.59
	0.8	0.779	97.33
	0.9	0.882	97.96

### Effect of interferents on oxidation peak potential

**Fig. 13:**
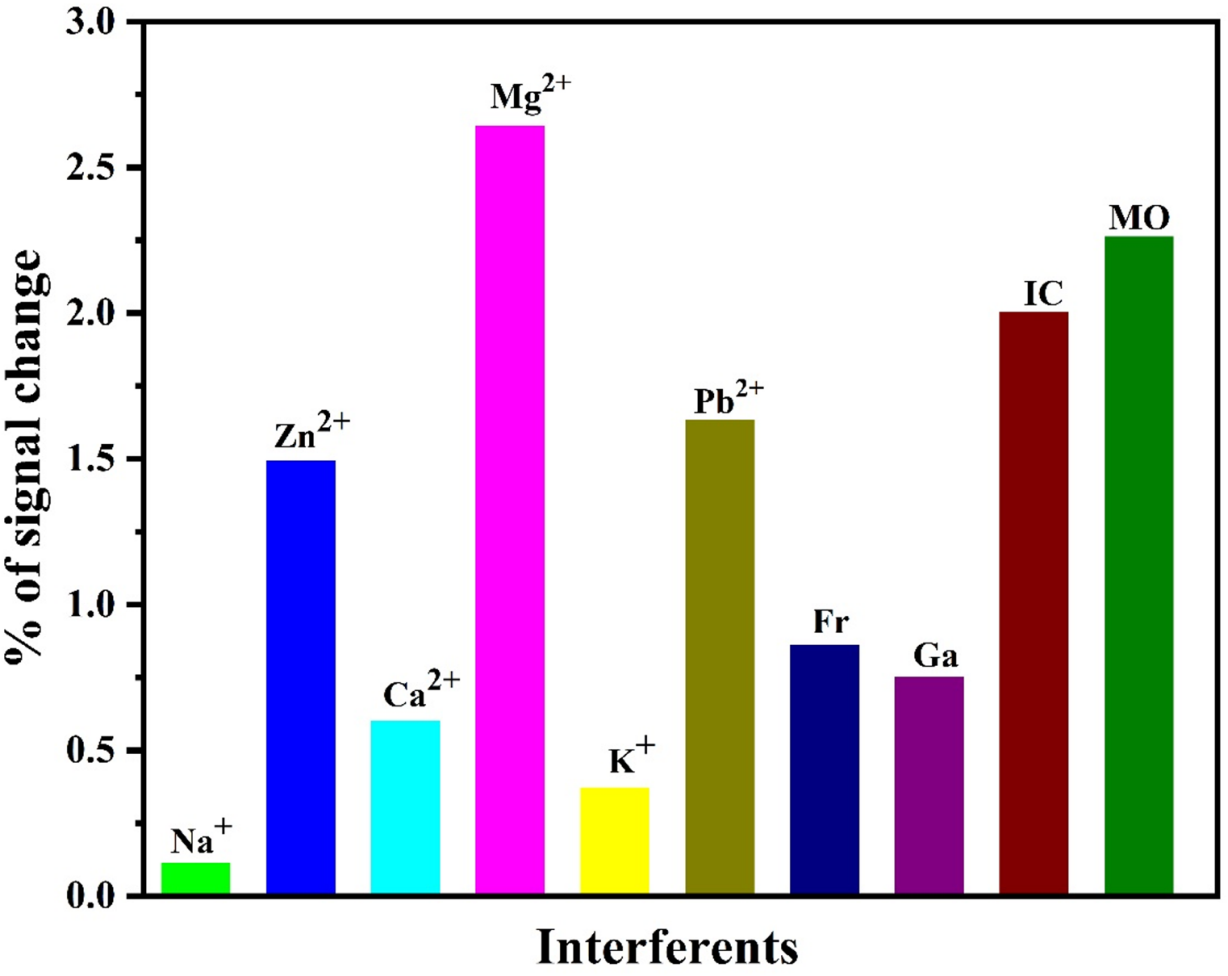
Graphical representation of potential variation in the presence of interferents in CV analysis of CM in PBS.

In the course of analysis, the presence of interferents might disrupt the current signals leading to inaccurate results. To analyze the effect of interfering species on the efficacy of SDSMCS, CV responses were documented for CM (1.0 mM) in PBS in the presence of interferents such as Na^+^, K^+^, Ca^2+^, Pb^2+^, Zn^2+^ and Mg^2+^ ions. In addition to these metal ions, the CV analysis was performed in the presence of simple sugars like fructose (Fr) and galactose (Ga) and dyes such as indigo carmine (IC) and methyl orange (MO). The observed results have been represented in Fig. 13 which demonstrates that the presence of interferents caused a slight variation of less than 5% in the peak potential, but no marked difference was noticed in the oxidation peak current values. This analysis authenticates the sensitivity of SDSMCS even in the existence of interfering metal ions, hence proving it as a reliable tool for the analysis of CM.

### Stability, repeatability and reproducibility of SDSMCS

The efficiency and reliability of the prepared SDSMCS were further evaluated by analyzing the attributes like repeatability, reproducibility and stability. CM (1.0 mM) was subjected to CV under optimum experimental conditions at the surface of five different SDSMCS. The current responses were observed to be almost similar with RSD of 4.95%, authenticating the high reproducibility of SDSMCS. Additionally, the current responses were recorded via CV at a single SDSMCS surface for five different solutions of 1.0 mM CM in PBS. The outcome demonstrated a great repeatability with RSD of 4.90%. Further, the current responses were documented with a gap of eight days (first day and the last day of work). The current maintenance noticed was 98.8%, hence substantiating the stability of SDSMCS for a significant time period.

## Conclusion

The fabrication of SDSMCS and its implementation in the assessment of CM analysis has been presented in this work. The morphological study of the bare and the modified sensors were performed by SEM which provided a clear distinction in the surfaces to verify the deposition of SDS on the sensor surface. At pH of 6.5, the anodic peak current of CM at SDSMCS was recorded as 96.38 µA at anodic peak potential of 0.5762 V. The adsorption-controlled oxidation process was found to be maximum with transfer of equal number of electrons and protons. The CV and DPV measurements demonstrated a steady increase of the anodic peak current with the varied CM concentration at optimal experimental conditions. The relevant data from the calibration plot provided a LOD and LOQ value of 0.009 µM and 0.031 µM (by CV) and 0.073 µM and 0.244 µM (by DPV), correspondingly. The practical application of SDSMCS was gauged and the outcome was remarkable with recovery rate exceeding 97% for food samples. The analysis substantiated the superior anti-interference detecting capacity of SDSMCS along with significant repeatability, reproducibility and stability attributes. Certain developments such as incorporation of nano materials and the use of bio-based surfactants in the analysis could further advance the performance and sustainability of the current work.

## Data Availability

The data that support the findings of this study are available on request from the corresponding author.
